# High clustering rates of multidrug-resistant *Mycobacterium tuberculosis* genotypes in Panama

**DOI:** 10.1186/1471-2334-13-442

**Published:** 2013-09-23

**Authors:** Samantha Rosas, Jaime Bravo, Franklin Gonzalez, Nora de Moreno, Joel Sanchez, Ronnie G Gavilan, Amador Goodridge

**Affiliations:** 1 Laboratorio Central de Referencia de Salud Pública, Instituto Conmemorativo Gorgas de Estudios de la Salud Pública, Vía Justo Arosemena, Panama, Panama; 2 Departamento de Microbiología, Facultad de Medicina, Universidad de Panamá, Vía Transístmica, Panama, Panama; 3 Centro de Biodiversidad y Descubrimiento de Drogas-Instituto de Investigaciones Científicas y Servicios de Alta Tecnología (INDICASAT-AIP), Ciudad Del Saber, Panama, Panama; 4 Centro de Biología Celular y Molecular de Enfermedades, Instituto de Investigaciones Científicas y Servicios de Alta Tecnología (INDICASAT-AIP), Ciudad Del Saber, Panama, Panama

**Keywords:** *Mycobacterium tuberculosis*, Multidrug-resistant tuberculosis (MDR-TB), DRE-PCR, (GTG)5-PCR, Spoligotyping

## Abstract

**Background:**

Tuberculosis continues to be one of the leading causes of death worldwide and in the American region. Although multidrug-resistant tuberculosis (MDR-TB) remains a threat to TB control in Panama, few studies have focused in typing MDR-TB strains. The aim of our study was to characterize MDR *Mycobacterium tuberculosis* clinical isolates using PCR-based genetic markers.

**Methods:**

From 2002 to 2004, a total of 231 *Mycobacterium tuberculosis* isolates from TB cases country-wide were screened for antibiotic resistance, and MDR-TB isolates were further genotyped by double repetitive element PCR (DRE-PCR), (GTG)5-PCR and spoligotyping.

**Results:**

A total of 37 isolates (0.85%) were resistant to both isoniazid (INH) and rifampicin (RIF). Among these 37 isolates, only two (5.4%) were resistant to all five drugs tested. Dual genotyping using DRE-PCR and (GTG)5-PCR of MDR *Mycobacterium tuberculosis* isolates revealed eight clusters comprising 82.9% of the MDR-TB strain collection, and six isolates (17.1%) showed unique fingerprints. The spoligotyping of MDR-TB clinical isolates identified 68% as members of the 42 (LAM9) family genotype.

**Conclusion:**

Our findings suggest that MDR *Mycobacterium tuberculosis* is highly clustered in Panama’s metropolitan area corresponding to Panama City and Colon City, and our study reveals the genotype distribution across the country.

## Background

*Mycobacterium tuberculosis* (*M. tuberculosis*) is the bacterium that causes tuberculosis (TB), which is the world’s leading infectious killer after human immunodeficiency virus (HIV). TB itself kills around 1.4 million people annually with virtually all deaths occurring in developing countries [1]. TB control has been impaired by the persistence of multidrug-resistant *M. tuberculosis* (MDR-TB). In 2011, Panama reported 1301 new pulmonary TB cases, including an estimated seven MDR-TB cases [2]. The estimated average prevalence of MDR-TB in Panama for 2011 was approximately 0.5%; however, a more detailed country-wide surveillance project is now underway [2]. Also, novel diagnosis and biomarker tests for monitoring the treatment response in MDR-TB cases are currently under evaluation.

MDR-TB case surveillance and control in Panama currently rely on conventional epidemiology tools. Presently, the health care system interviews MDR-TB-affected patients to collect demographic characteristic information, as recommended by WHO [3]. In addition, information about past history of TB and treatment, type and extent of disease, concomitant medical illnesses and substance abuse, among others risk factors, is also collected. However, investigation of these MDR-TB patients’ contacts remains limited, and thus, conventional epidemiology findings are unable to prove true links between most MDR-TB cases [4,5]. Consequently, the National TB Program (NTBP) continues to invest enormous time and effort to determine transmission dynamics within communities in order to implement appropriate strategies for TB and MDR-TB control and prevention.

For more than a decade, researchers have demonstrated the use of genomic markers to study TB and MDR-TB epidemiology as a useful strategy for understanding TB transmission dynamics [6]*.* Rapid and inexpensive genotyping based on PCR assays, such as double repetitive element PCR (DRE-PCR) and (GTG)5, have proven to be useful in determining the genetic relationships and epidemiology of TB and MDR-TB within vulnerable communities; these genotyping techniques identify and track individual *M. tuberculosis* strains [7-13]. Similarly, spoligotyping is a well-established commercial method that provides worldwide standardized information about *M. tuberculosis* isolates’ family lineage background [14-17]. However, despite these laboratory improvements, data on the transmission and distribution of MDR *M. tuberculosis* strains within Panama remain scarce. Therefore, our study aimed to characterize the distribution of MDR *M. tuberculosis* isolates from the Republic of Panama collected between 2002 and 2004 using DRE-PCR and (GTG)5-PCR in a dual genotyping strategy and spoligotyping.

## Methods

### MDR *M. tuberculosis* clinical isolates

*M. tuberculosis* isolates from 231 suspected multidrug-resistant pulmonary TB cases were collected between January 2002 and December 2004 from local laboratories at nine different health centers and hospitals located throughout the Republic of Panama. Because the strain collection was performed as part of the Panamanian standard of patient care for TB diagnosis and control, this study was not submitted to ethical committee evaluation. The identification of *M. tuberculosis* isolates was performed using biochemical tests including niacin, nitrate and catalase tests [18]. All isolates were evaluated for antibiotic resistance on Lowenstein-Jensen (LJ) medium according to the Cannetti proportion method [19]. Briefly, a suspension of *M. tuberculosis* clinical isolate was placed in an LJ tube with and without drugs. *M. tuberculosis* colony forming units (CFUs) were enumerated in the drug-free medium (control tubes) and in the tubes with anti-tuberculosis drugs. Each LJ media tube contained a single antibiotic at the following concentrations: 0.2 mg/ml isoniazid (INH), 40 mg/ml rifampicin (RFP), 4 mg/ml streptomycin (SM), 2 mg/ml ethambutol (EMB) and 2 mg/ml paraminosalicylic acid (PAS). The resistance percentage was calculated by multiplying the ratio of the CFUs in media with drugs to the CFUs in media without drugs by 100. No growth in the LJ medium with anti-tuberculosis drugs indicated a sensitive strain. *M. tuberculosis* isolates that were resistant to at least rifampicin and isoniazid were classified as MDR *M. tuberculosis* and stored in 10% skim milk (Difco, USA) at -70°C until further DNA extraction and genotyping.

### DNA isolation from MDR *M. Tuberculosis* clinical isolates

DNA was extracted from *M. tuberculosis* isolates with Chelex^®^ using a method described previously [20,21]. Briefly, two or three colonies were inactivated at 80°C for 30 min in Tris-EDTA (10 mM Tris, 1 mM EDTA, pH 7.4) buffer and then immediately placed on ice. Then, the bacteria were centrifuged at 10,000 rpm for two min. The supernatant was then discarded and the pellet resuspended in 500 μl of 5% Chelex-100. The suspensions were vortexed for a few seconds and incubated at 56°C for 20 min, boiled at 100°C for 10 min. and then placed on ice for two min. and centrifuged at 13,000 rpm for five min. The supernatant containing the DNA was stored at -20°C.

### DRE-PCR genotyping

Isolated DNA from MDR *M. tuberculosis* isolates was analyzed with a modified DRE-PCR protocol [8,10]. The PCR assays were performed in a 25-μl reaction volume containing three μl of the extracted DNA, Go-Taq Green Master Mix (Promega Corporation, USA) supplemented with 4.5 Mm MgCL2, 6% dimethyl sulfoxide (DMSO), 1 U Taq DNA polymerase (Bioselec S.A., Mexico D.F.) and the four primers Ris1, Ris2, Pntbl and Pntb2 at 25 pmol each as previously described [10]. The PCR was performed as described by Friedman et al.*,* including an additional ramp of +0.7°C per second between the denaturation and annealing segments [10]. PCR products were analyzed by electrophoresis on 2% agarose gel and visualized with ethidium bromide in a gel documentation system (Ultra-Lum Omega System, USA).

### (GTG)5-PCR genotyping

DNA fingerprinting of MDR *M. tuberculosis* was carried out using a modification of previously described protocols [12,22]. Briefly, DNA samples from MDR *M. tuberculosis* strains were subjected to rep-PCR using the (GTG)5 primer (5′-GTGGTGGTGGTGGTG-3′). The PCR assays were performed in a 50-μl reaction volume containing 10 ng of genomic DNA, one unit of Taq DNA polymerase (Roche, Mannheim, Germany), 0.8 mM of each primer (Sigma-Aldrich, St. Louis, MO), 1.5 mM MgCl2 and 200 μM of each deoxynucleoside triphosphate (Roche, Mannheim, Germany). PCR cycling conditions consisted of initial activation at 94°C for five min, followed by 35 cycles of a three-step PCR program (94°C for 45 seconds, 40°C for one min. and 65°C for 10 min.) and a final extension at 65°C for 20 min. PCR products were analyzed by electrophoresis on 1.2% agarose gel and visualized with ethidium bromide.

### Spoligotyping genotyping

Spoligotyping was performed on genomic DNA using the standard method described by Kamerbeek et al. [14]. Spoligotypes were reported in standard international type number (SIT) and octal code numbers were obtained from the SPOLDB4.0 [23].

### Data analysis

The fingerprints obtained using both DRE-PCR and (GTG)5-PCR were analyzed using Bionumerics Software version 6.6 (Applied Maths, Sint-Martens-Latem, Belgium). Band patterns were evaluated using the Dice similarity coefficient with a 4% band tolerance and 1% of optimization. Dendograms were constructed using the unweighted pair group method with arithmetic mean (UPGMA). Related strains in a cluster were defined as having more than 90% of homology. The similarity between (GTG)5-PCR and DRE-PCR was calculated using the Pearson correlation coefficient (r value) using Bionumerics Software.

## Results

**Figure 1:**
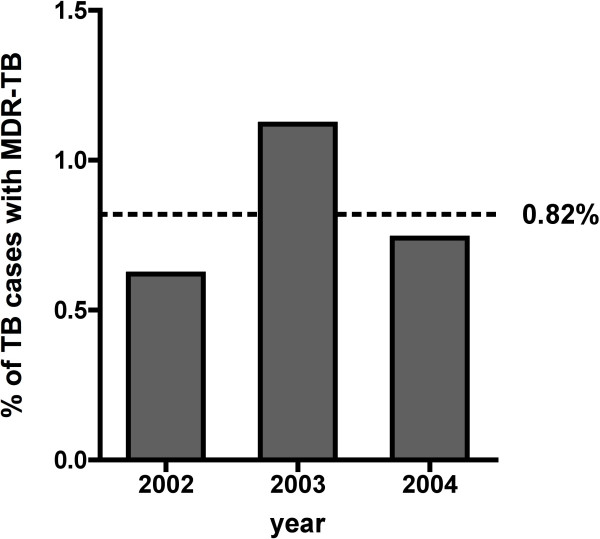
**Proportion of MDR-TB cases in Panama from 2002 to 2004.** Country-wide *M. tuberculosis* clinical isolates were evaluated by the Canetti drug susceptibility test between 2002 and 2004. Thirty-seven isolates were multidrug-resistant (MDR). The bars show the percentage of MDR *M. tuberculosis* strains per year. The dotted line indicates the countrywide average proportion of TB cases with MDR-TB for the three-year period.

From 2002 to 2004, the Panamanian NTBP reported a total of 4507 new pulmonary TB cases country-wide. Of those new cases, 231 *M. tuberculosis* clinical isolates were sent to Laboratorio Central de Referencia de Salud Pública de Instituto Conmemorativo Gorgas de Estudios de la Salud (LCRSP-ICGES) for drug susceptibility testing (DST). At least 69% (160) of these isolates were originally obtained from the metropolitan region, including San Miguelito, Panama Este, Panama Oeste and Colon City. The DST results revealed monoresistance to INH (29.0%), RFP (30.7%), SM (22.1%), EMB (8.2%) and PAS (0.9%) in the collection of 231 *M. tuberculosis* isolates. Thirty-seven isolates collected between 2002 and 2004 demonstrated resistance to at least INH and RFP, and these isolates were defined as MDR *Mycobacterium tuberculosis.* Thus, an average prevalence for the three-year period is estimated at 0.82% of the total national TB cases reported (Figure 1). These 37 MDR *M. tuberculosis* isolates were not distributed uniformly across Panama. Colon City itself accounted for 40% of the MDR-TB cases, whereas Chiriqui and the Metropolitana region accounted for 23% and 20%, respectively (Table 1). Within these 37 MDR-TB isolates, 76% (28 isolates) were resistant to INH, RIF and SM. Only 5.4% (two isolates) of the MDR-TB isolates showed resistance to all five drugs

**Table 1:** **Resistance and genotype profiles of MDR *M. tuberculosis *clinical isolates in Panama 2002-2004**

**Region**	**N (%)**	**Resistance profiles**	**DRE-(GTG)5 **c**lusters**	**Spoligo international types**
Colon	15 (40.5)	a, b, c	A, B, G, K	42, 46, 68
Chiriqui	10 (27.0)	a, b, c	A, C, D, F, H, I, J	42, 119, unknown
Metropolitana	5 (13.5)	a, b	B, C	42, 68
Panama Oeste	2 (5.4)	b	L, E	46
San Miguelito	2 (5.4)	a, b	B, M	42, 1366
Panama Este	1 (2.7)	b	L	119
Changuinola	1 (2.7)	a	H	17
Veraguas	1 (2.7)	a	N	42

Note: Resistance profiles are classified as follows: a) INH, RFP; b) INH, RFP, SM; c) INH, RFP, SM, EMB.

**Figure 2:**
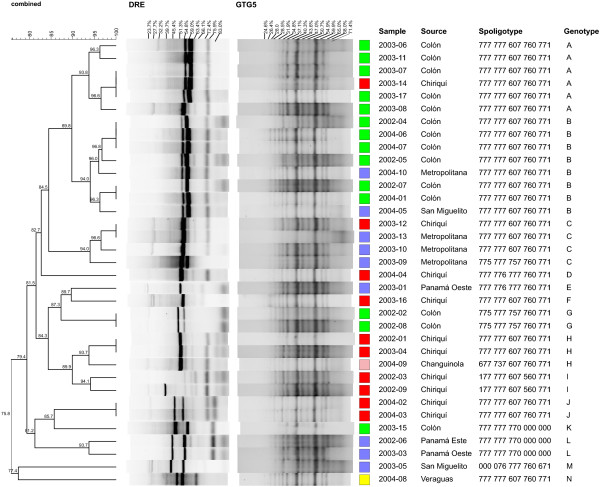
**Combined dendogram based on DRE-PCR and (GTG)5 genotyping.** Genomic DNA was extracted from MDR *M. tuberculosis* strains and amplified by multiplex (GTG)5-PCR and DRE-PCR. Dendograms were built using the Dice coefficient at 4% band tolerance and 1% optimization. The correlation for each band is shown at the top of the figure.

The combined analysis of DRE-PCR and (GTG)5-PCR showed a high clustering of MDR *M. tuberculosis* clinical isolates in the Colon and Metropolitana regions of Panama. Fourteen different DRE-PCR patterns were obtained with a range of two to eight bands. In contrast, (GTG)5-PCR showed six different patterns with a range of eight to fifteen bands. Two MDR *M. tuberculosis* isolates did not amplify and were excluded from the clustering computer analysis (Figure 2). A comparison between both methods revealed only a 15.3% correlation (Pearson *p* = 0.0424). Therefore, we assigned a genotype to each strain using both methods in a dual typing strategy. The dual genotyping identified Cluster A, which consisted of six out of the 35 isolates (17.1%). In this cluster, four strains were isolated from Colon City and one was isolated from Chiriqui. Cluster B included eight isolates (22.9%), six from Colon City, one from the Metropolitana region and one from San Miguelito. Cluster C consisted of four isolates (11.4%) from our MDR *M. tuberculosis* set. This cluster was composed of three isolates from the Metropolitana region and one from Chiriqui. Cluster G included 5.7% (2/35) of MDR *M. tuberculosis* isolates, both from patients from the Colon region. Cluster H contained three out of the 35 isolates (8.6%). Clusters I and J consisted of two isolates each (5.7%), of which all four MDR *M. tuberculosis* cases were from the Chiriqui region. Finally, Cluster L included two isolates from Panama Este and two isolates from Panama Oeste. Six MDR *M. tuberculosis* isolates (17.1%) were not grouped into the clusters described above (Figure 2).

The spoligotyping analysis typed almost 68% of MDR *M. tuberculosis* isolates as members of the 42 (LAM9) family. Remaining MDR-*M. tuberculosis* isolates were classified as spoligo international type (SIT) numbers 68, 46, 119, and 1366 according to the SPOLDB4.0 International Database (Table 1) [23]. We identified two MDR *M. tuberculosis* isolates that were not annotated in this database.

## Discussion

During the 2002–2004 period, a collection of 231 *M. tuberculosis* isolates were identified to have monoresistance to INH (29.0%), RFP (30.7%), SM (22.1%), EMB (8.2%) or PAS (0.9%). Thirty-seven (0.82%) of these isolates were recognized as MDR *M. tuberculosis* because they were resistant to both INH and RFP drugs. Among these MDR *M. tuberculosis* isolates*,* two (5.4%) were resistant to all five drugs. Our study results show that nearly 83% of these MDR *M. tuberculosis* cases were grouped within Panama City and Colon City, indicating a higher clustering rate compared to other cities in the Latin American region, such as 39.4% reported in Havana and 55.3% reported in Rio de Janeiro [8,24]. Specific molecular cluster patterns were found to be prevalent in Colon, San Miguelito and the Metropolitana region within the Republic of Panama. Colon City harbored the two largest clusters identified in this strain collection (Cluster A and B). Similarly, the MDR *M. tuberculosis* strains recovered from the Chiriqui region were clustered. These findings suggest that these MDR *M. tuberculosis* strains were spread rapidly during the period from 2002 to 2004.

Our data indicate that the Colon region has the highest number of clustered MDR *M. tuberculosis* isolates. One plausible explanation is the high TB incidence rate, which reached nearly 100/100,000 inhabitants in Colon during the study period [25]. In addition, the HIV-TB coinfection rate was up to 40% during those years. These two factors combined with Colon’s lower socioeconomic status, extreme poverty levels and overcrowded living conditions might favor the transmission of MDR *M. tuberculosis* strains. New collaborative interventions to reinforce the TB-HIV response are currently being implemented by health authorities, INDICASAT-AIP and University Research Co., LLC (URC-CHS) to limit this situation, not only in Colon but across the entire Republic of Panama.

The overall high clustering rate found in our study indicates the possibility that patients belonging to a cluster might have been infected with an *M. tuberculosis* strain that easily became resistant. Another possible explanation is that an MDR *M. tuberculosis* mutant strain was selected by mono-therapy or irregular treatment compliance [26]. The exact reason for our findings remains to be explored by further combining bacterial genomics and patient epidemiological data. A recent study demonstrated the distribution of point mutations conferring resistance in a collection of MDR *M. tuberculosis* isolates from Panama using a multiplex PCR platform [27]. This study revealed that the most frequent point mutation is located in codon 315 of the KatG gene. However, it is expected that MDR M. tuberculosis isolates with a KatG gene mutation are likely to be clustered [28]. Molecular epidemiology studies using whole genome sequencing or VNTR-MIRU are now required to determine the extent of MDR-TB transmission and monitor the expansion of this infection, especially in the Colon region [29,30].

The study of MDR-TB cases in Panama currently uses conventional epidemiology tools. The contact investigations for these MDR-TB cases remain limited, and conventional epidemiology results are unable to prove true links between most MDR-TB cases [4,5]. The use of PCR-based genotyping tools, such as those used in the present study, might be helpful in conducting the epidemiological contact investigation of MDR-TB cases in Panama [8,10,11]. Although DRE-PCR showed a poor correlation with (GTG)5 patterns in our study, a dual genotyping strategy using both techniques increased the accuracy when analyzing our MDR *M. tuberculosis* strain collection. Because the cost of PCR and primers has decreased, this dual strategy might be useful in settings without access to more expensive, cutting edge technologies.

The reasons for the persistence of MDR *M. tuberculosis* strains in Panama remain unknown. One explanation might be stochastic introduction resulting from migration for the Panama Canal construction at the end of the nineteenth century. Other plausible explanations include malnutrition, lack of adherence to anti-TB therapy and/or the intermittent availability of drugs. As depicted in our study, the spoligotype 42 (LAM9) was present in almost 68% of the MDR *M. tuberculosis* isolates studied. Despite Panama’s geographical proximity to Valle de Cauca, Colombia, we did not find Beijing genotypes in the MDR-TB isolates from Panama [31]. Similarly, other studies in America have found wider genetic diversity when genotyping MDR *M. tuberculosis* strains using spoligotyping [15-17]. Our sample collection was not created under a systematic national MDR surveillance program; however, the findings indicate a need to redirect efforts in order to understand the transmission dynamics of MDR *M. tuberculosis* strains.

## Conclusion

The findings presented here reveal that most MDR *M. tuberculosis* strains isolated from Panama City and Colon City are highly clustered. Our study confirmed the clustering of a limited number of MDR-TB cases using an inexpensive dual genotyping strategy; this strategy could be extended to countrywide MDR surveillance to gain a better understanding of MDR-TB transmission dynamics and contribute to MDR-TB control in the short term.

### Ethical approval

The strain collection was performed as part of the Panamanian standard of patient care for TB diagnosis and control; thus, the study was not subject to ethical committee evaluation.

## Abbreviations

TB: Tuberculosis; MDR: Multidrug-resistant; MDR-TB: Multidrug-resistant tuberculosis; NTBP: National tuberculosis control program; DRE-PCR: Double repetitive element – polymerase chain reaction.

## Competing interest

The authors declared that they have no competing interest.

## Authors’ contributions

SR, JB and FG performed the drug susceptibility tests. SR, AG and JS performed the genotyping. JS and RG performed the software-based fingerprint analysis. All authors wrote, edited and approved the manuscript.

## Pre-publication history

The pre-publication history for this paper can be accessed here:

http://www.biomedcentral.com/1471-2334/13/442/prepub
